# Stressful life events in electronic health records: a scoping review

**DOI:** 10.21203/rs.3.rs-3458708/v2

**Published:** 2023-12-13

**Authors:** Dmitry Scherbakov, Abolfazl Mollalo, Leslie Lenert

**Affiliations:** Biomedical Informatics Center, Department of Public Health Sciences, Medical University of South Carolina; Biomedical Informatics Center, Department of Public Health Sciences, Medical University of South Carolina; Biomedical Informatics Center, Department of Public Health Sciences, Medical University of South Carolina

**Keywords:** EHR, electronic health records, life change events, negative life events, social determinants of health, stressful life events

## Abstract

**Objective.:**

Stressful life events, such as going through divorce, can have an important impact on human health. However, there are challenges in capturing these events in electronic health records (EHR). We conducted a scoping review aimed to answer two major questions: how stressful life events are documented in EHR and how they are utilized in research and clinical care.

**Materials and Methods.:**

Three online databases (EBSCOhost platform, PubMed, and Scopus) were searched to identify papers that included information on stressful life events in EHR; paper titles and abstracts were reviewed for relevance by two independent reviewers.

**Results.:**

557 unique papers were retrieved, and of these 70 were eligible for data extraction. Most articles (n=36, 51.4%) were focused on the statistical association between one or several stressful life events and health outcomes, followed by clinical utility (n=15, 21.4%), extraction of events from free-text notes (n=12, 17.1%), discussing privacy and other issues of storing life events (n=5, 7.1%), and new EHR features related to life events (n=4, 5.7%). The most frequently mentioned stressful life events in the publications were child abuse/neglect, arrest/legal issues, and divorce/relationship breakup. Almost half of the papers (n=7, 46.7%) that analyzed clinical utility of stressful events were focused on decision support systems for child abuse, while others (n=7, 46.7%) were discussing interventions related to social determinants of health in general.

**Discussion and Conclusions.:**

Few citations are available on the prevalence and use of stressful life events in EHR reflecting challenges in screening and storing of stressful life events.

## Introduction

Stressful events that individuals experience during life-course have a wide range of consequences for both mental and physical health [1–3]. At the same time, electronic health records (EHR) often lack information about these events [4]. Even so, care from a whole person should include support for these events within clinical encounters.

By stressful life events in this study, we mean highly stressful events that a person views as undesirable, unplanned and possibly traumatic. This definition is reflected in Encyclopedia of Quality of Life and Well-Being Research [5]. Common stressful events include going through abuse, loss of financial means to support oneself, and death of relatives or an intimate partner. A well-established reference collection of such events with an added scale of significance of each event is called the ‘Social Readjustment Rating Scale’, introduced in 1967 and later revised by several authors [6, 7]. However, many events in these scales, such as getting married (as opposed to forced or child marriage), typically don’t have the qualities of being unplanned, undesirable, and stressful at the same time. For the same reason, other normative events, such as immigration and household moves (as opposed to forced displacement and eviction), do not fit into our definition of stressful life events, while acknowledging that for some family members these events can be unexpected and undesirable at the same time.

In the context of public health, stressful life events can be viewed as a major personal shock experienced in one of the domains of social determinants of health (SDOH): education, employment, healthcare, social support, neighborhood and living environment [8]. However, life events are personal psychosocial events that can be potentially missed from an SDOH domain documentation perspective. To give a concrete example, the Committee on the Recommended Social and Behavioral Domains and Measures for Electronic Health Records recommends assessment of whether a patient is employed or unemployed/laid-off, but a related stressful event – being fired or laid-off from a job – falls in the recommended measures under the category of stress [9]. The Committee proposed to assess stress using a single question which, arguably, is better suited for measuring current stress than stressful life events (“Stress means a situation in which a person feels tense, restless, nervous, or anxious, or is unable to sleep at night because his/her mind is troubled all the time. Do you feel this kind of stress these days?”). The experience of unexpected job loss is distinct from daily stress and from the state of being unemployed and its stressful effects might last long after employment is regained. In general, it is reasonable to differentiate stressful life events from daily and chronic stressors with the former having a more acute and discrete nature [10].

There are additional reasons to consider life events separately from SDOH or as a distinctive group or dimension inside SDOH. Information about such events, in our opinion, doesn’t have a commonly mentioned limitation of SDOH as part of EHR – the practical inability to act upon them in a clinical setting [11, 12]. On the contrary, stressful events may have a more direct clinical utility. For example, care providers may consider them when assessing symptoms or refer patients to mental health specialists. Considering traumatic life events can be critical in proactive suicide detection [13]. On the other hand, it is worth considering events occurring in a wider social network of a patient. For instance, a spouse experiencing job loss also can be a perpetrator of family violence [14].

Some stressful personal events, such as the death of a beloved family pet, are difficult to express within the SDOH framework; others such as experiencing gender dysphoria or being arrested, tend to be missed because they are considered “rare events” [9, 15]. Arguably, life events as individual experiences can be better approached using an anthropological paradigm [16]. Nevertheless, a range of stressful life events is incorporated as part of SDOH codes in the ICD-10 edition [17]. This approach, while having limitations noted above, is beneficial for creating a more coherent psychosocial portrait of the patients [18].

The incorporation of SDOH into EHR is rapidly evolving [12, 19, 20], and stressful events, similar to SDOH factors, may be underrepresented in EHR. Practical challenges surround collection of information related to ICD-10 Z-codes [21]. Several researchers noted that much more information about SDOH can be retrieved from clinical notes in EHR compared to structured EHR fields [22, 23]. On the other hand, research shows that clinical notes, such as social history, usually do not provide a comprehensive view of the social situation of a patient and tend to miss significant life experiences [24].

To our knowledge, no previous study has examined how stressful life events are stored and used in EHR. We have opted for the scoping review following the guidelines outlined by Arksey & O’Malley [25]. Our pilot citation search indicated that this area of research has substantial gaps. Moreover, we identified a range of papers, including gray literature, and various study designs focusing on life events. Thus, the goal of this scoping review is to review and summarize the literature on (1) what types of stressful life events are captured in EHR, (2) how they are stored in EHR, (3) what tools are proposed to extract such events from EHR, and (4) how such life events from EHR are used in clinical workflows and further research on patient risk factors and outcomes.

## Methods

The research protocol for this study was created and revised following the recommendation of Preferred Reporting Items for Systematic Reviews and Meta-analysis Protocols extension for Scoping Reviews (PRISMA-ScR) and updated JBI guidance for the conduct of scoping reviews [25–28]. The protocol is available as a supplement (Supplementary File S1).

To be included in the review, references had to focus on EHR and either discuss stressful life events in general or focus specifically on some types of the events, such as the death of a relative or marital dissolution. All English-language publications were considered, including dissertations and conference abstracts, reviews, and all other types of literature.

Citations were excluded from full-text screening for several reasons:

1. The citation was focused on daily stress rather than stressful life event; one exception is made for minority stressors, where we tended to include all stressors encountered, including the ones, which can be considered daily stress (for example, gender discrimination, sexism, racial discrimination, etc.). In many cases, discrimination is accompanied by a series of stressful and traumatic episodes (for example, being denied a mortgage or a job) [10]. Overall, our approach to stress in minorities was more inclusive because we didn’t have a reference scale of stressful life events for minority populations, similar to Social Readjustment Rating Scale [7].
2. The citation discussed normative life course events such as employment, unemployment, marriage, childbirth, unless there is an indication that they are treated as stressful or traumatic life events (and not as demographic status variables or normative life events);
3. Physical conditions such as diseases, injuries, and pregnancy were not considered as stressful life events, unless they were explicitly considered as such.

The first search was conducted in June of 2023, using three databases: PubMed, Scopus, and all sources in EBSCOhost platform. We included these sources to cover a broad range of citations, including gray literature from medical informatics to social psychology. The list of search terms was compiled based on the Social Readjustment Scale and Adolescent Life Change Event Scale [7, 29], while at the same time including general terms like “stressful events”, “life-change events”, and similar to capture stressful life events which we didn’t have a notion about beforehand. Search query strategy for one of the databases developed with the assistance of an experienced librarian (Supplementary Appendix S2). In August of 2023, an additional search was initiated with the same databases, but other set of search terms to fetch more articles reflecting the clinical utility of stressful life events in EHR (Supplementary Appendix S3). In November 2023 we conducted an additional search to include stressful events for minority populations. This search was conducted using a list of minority stressors compiled from a range of publications [28, 30–33] (Supplementary Appendix S4).

Manual references search was completed for included citations following full-text screening. All search results were exported to Covidence online software, where the remaining review and extraction took place.

The screening was independently performed by two authors (DS and AM), and the data charting form was completed by one author (DS) with another author (AM) supervising the process and double-checking the entries. Disagreements were resolved by reaching a consensus during discussions.

The data charting form and categories for extraction were developed and tested with the help of an experienced EHR consultant and stress recovery expert. Categories were selected to answer main research questions: what types of stressful life events are considered by authors of the citation; how stressful life events are stored in EHR (structured, unstructured, or linked data); if the citation was focused on EHR, but stressful events were not stored in EHR, where were they sourced from (survey, external dataset); what is the focus of the publication (for example, finding a statistical association or extracting life events from unstructured data). Additionally, for the studies focused on the statistical association between life events and other variables: what was the nature of outcome variables (physical health, mental health, health behavior, borderline/mixed). For citations focused on clinical utility, details on clinical intervention were extracted. In addition, first author, year of each publication, the region where study was conducted (or, if it can’t be determined, where the first author’s affiliated organization is located) were identified. Additional details on extracted categories are provided in the protocol (Supplementary File S1).

After extracting information from the studies, we proceeded with narrative synthesis of findings which was accompanied by a summary table, a table with full information on extracted categories, and an array of column plot figures to numerically summarize the categories. We also generated a map showing geospatial distribution of the regions where reviewed studies were conducted.

## Results

**Figure 1:**
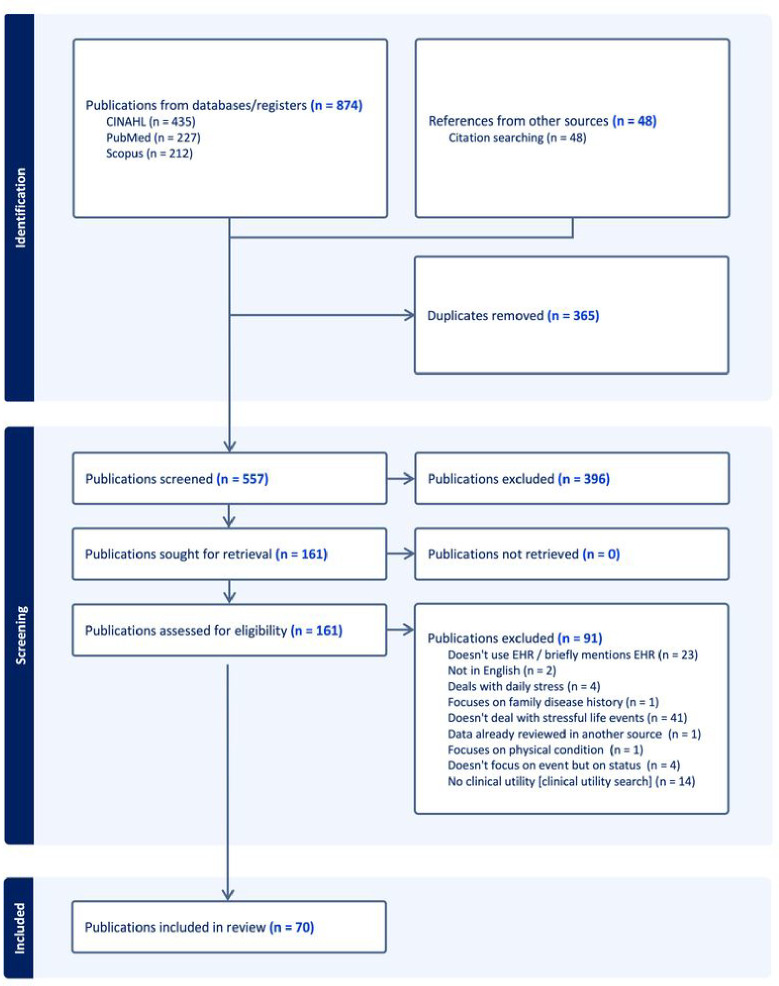
Flow diagram of citation selection process

Figure 1 illustrates the PRISMA article selection process. Initially, 557 unique citations were identified from searches of electronic databases and reviewing article references (snowballing). After the title and the abstract screening, 396 were excluded, with 161 full-text publications to be retrieved and assessed for eligibility. Of these, 91 did not meet the inclusion criteria. The remaining 70 citations were considered eligible for inclusion in this review.

**Figure 2:**
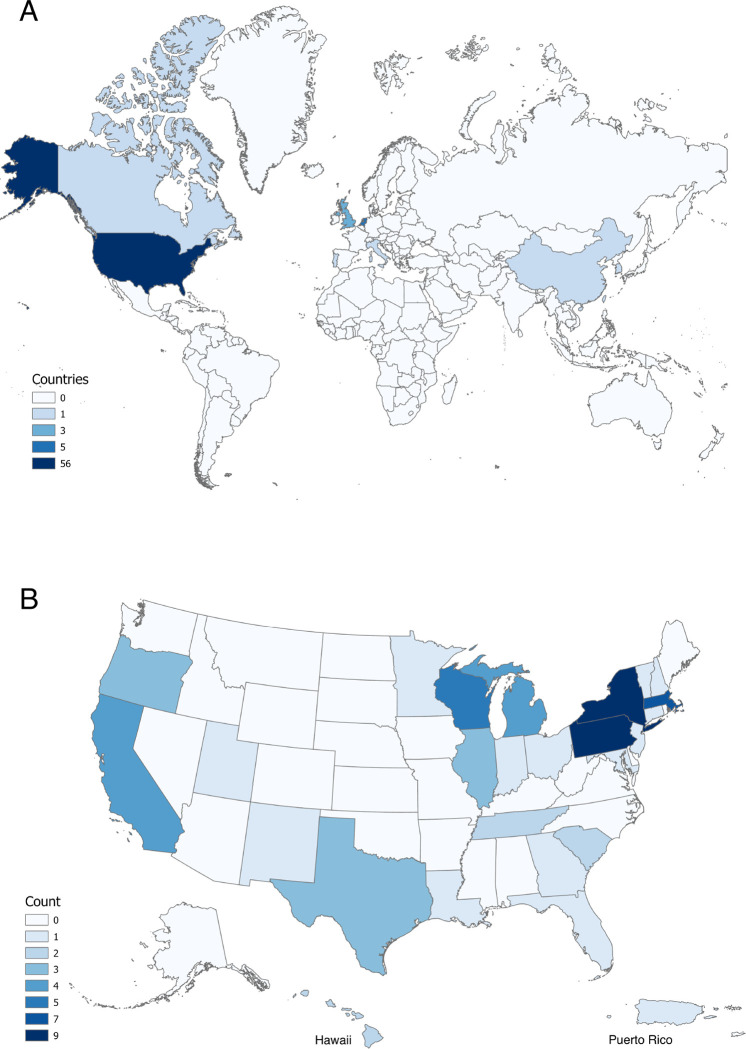
Citations by place of origin. A: By country; B: By US state.

Most citations focusing on stressful life events and EHR were coming from the US (n=56, 80%), Netherlands (n=5, 7.1%) and UK (n=3, 4.3%). Canada, China, Italy, Portugal, Puerto Rico, and South Korea were represented by one publication. Reference characteristics by country of origin and US states statistics are provided in Figure 2.

Most citations can be categorized into five following areas by their focus (a single citation can span through several focus areas):

1. Finding statistical dependency, in which one of the variables is stressful life event(s) (n=36, 51.4%);
2. Clinical utility of stressful life events in EHR (n=15, 21.4%);
3. Extracting events from EHR (n=12, 17.1%);
4. Discussing issues of storing life events in EHR (n=5, 7.1%), such as privacy;
5. Proposing new EHR design, template, or feature to facilitate stressful life events capturing (n=4, 5.7%).

**Figure 3:**
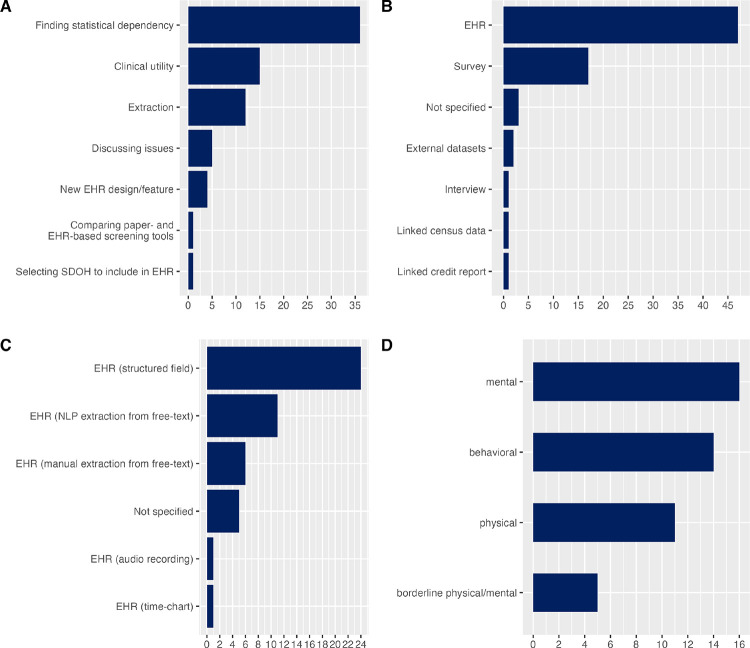
A: Citations by area of focus; B: Citations classified by source of stressful life events; C: Citations by EHR data types and methods of extraction; D: Outcome variables in statistical studies by their area.

The remaining two publications are focused as follows. One study [34] compared paper-based and EHR-based screening tools and found that capturing adverse life events improved significantly when using specialized tools in EHR: 77% of EHR had information on such events compared to 33% when using older paper-based tools. One more citation [9] is the already mentioned work of the Committee on the Recommended Social and Behavioral Domains and Measures for Electronic Health Records, which debated what measures should be considered for stressful life events and decided that a general measure of stress would be sufficient. Figure 3A depicts the focus areas of all included articles.

Most of the citations (n=47, 67.1%) discussed or utilized life events stored in various formats in EHR. However, a significant number of citations (n=17, 24.3%) obtained life events via external surveys, while health outcomes were extracted from EHR. These citations were focused on statistical analysis. Another publication linked a credit history report to identify serious financial events in patients’ lives, such as bankruptcy [35]. One more citation utilized the interview format for events identification and compared them to what was documented in EHR and found that health records “generally failed to include social contexts salient to patients” [36]. One citation [37] discussed issues of using external data sources linked to EHR for capturing “financial, legal, life event and sociodemographic data” to improve suicide screening models. Another citation used public databases to identify people who survived mass shootings [38]. One study investigated the impact of the 2008 economic crisis on different health outcomes using the unemployment data from the census [39]. Figure 3B demonstrates breakdown of the articles by source of stressful life events.

Among 47 publications that were focused on EHR as a source of stressful life events, structured data on events was used in more instances (n=24, 51.1%) than unstructured data (n=18, 38.3%). One additional paper [40] discussed a time chart feature inside EHR showing correlations between life events and body mass index. In 18 citations using unstructured data natural language processing (NLP) was the most popular method for extraction (n=11, 61.1%), followed by manual abstraction (n=6, 33.3%), audio recording was used as mode of storage of life events in one citation [41]. Figure 3C illustrates the data types for stressful life events in EHR.

Among 36 studies that were focused on finding statistical dependency, the health outcome under question was most often in the domain of mental health (n=16, 44.4%), such as diagnosis of post-traumatic stress disorder, and anxiety; followed by behavioral outcome variables, such as violent and self-harmful behavior (n=14, 38.9%), and physical health outcomes (n=11, 30.6%). Few studies (n=5, 13.9%) used dependent variables from the borderline area spanning across physical and mental health, such as in the case of chronic pain. Figure 3D summarizes observed outcome variables.

**Figure 4:**
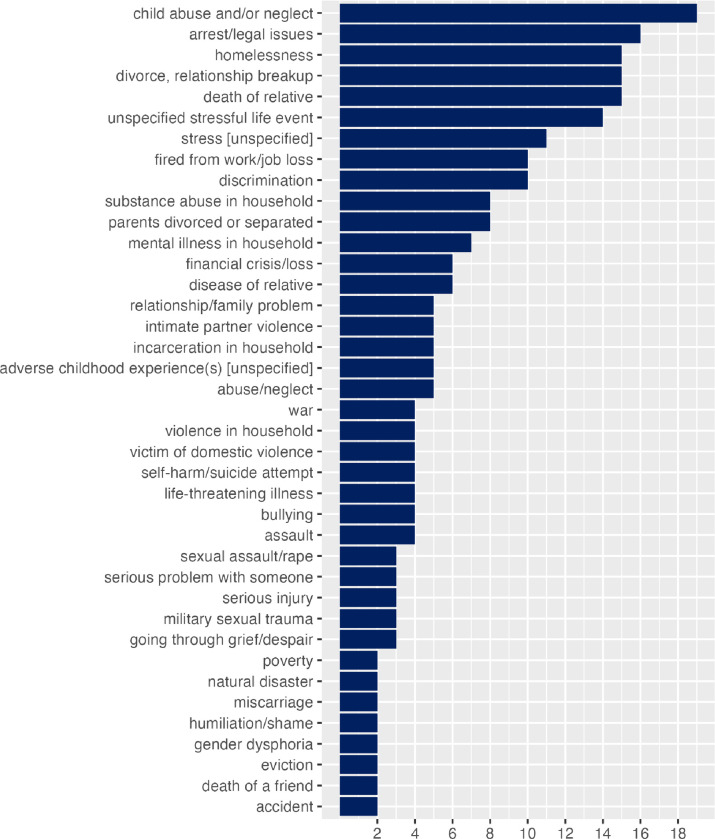
Stressful life events by number of citations in which they are mentioned (excluding events mentioned by only one citation)

There were recurring stressful life events seen across multiple citations, such as child abuse and/or neglect, arrest/legal issue, divorce or relationship breakup, death of relative, loss of job, homelessness. Different types of stressful life events, mentioned in at least two citations, are presented in Figure 4. A sizable number of events were rare, they didn’t appear in more than one citation: abandoned by parent, acculturative stress, captivity, child marriage, disappearance of family member, forced marriage, genital mutilation/cutting, life-threatening event, mass shooting, miscarriage, parental behavioral problems, pregnancy, psychological trauma, sex trafficking in childhood, sexism, sexual exploitation, something valuable lost or stolen, starvation, terrorism, took drugs as a child, unwanted pregnancy, victimization, weight stigma, witnessing somebody’s injury or death.

Child abuse and/or neglect was the most common type of stressful life event mentioned in citations (n=19, 27.1%). A sizable number of publications (n=14, 20%) considered a serious life event but did not specify what event it was. An example of this approach is a survey question “After the age of 17, did you experience any major upheaval that you think may have shaped your life or personality significantly?” [42]. Unspecified stress was used in a sizable portion of citations (n=11, 18.3%), a common question to measure it was “Stress means a situation in which a person feels tense, restless, nervous, or anxious, or is unable to sleep at night because his/her mind is troubled all the time. Do you feel this kind of stress these days?”. This question is used in various popular screening tools such as PRAPARE [43–45].

Among the 15 articles that analyzed clinical utility, a little less than a half (n=7, 46.7%) were focused on child abuse and neglect. These publications discussed several clinical processes which are triggered when such events are detected, such as alert in EHR, starting an abuse-related order set, and reporting to child protection agencies. One study introduced cognitive behavioral therapy to pregnant Latinas and African Americans experiencing discrimination and acculturative stress to reduce pregnancy complications and postpartum depression [46]. The remaining group of articles (n=7, 46.7%) touched on the topic of possible clinical workflow alterations based on detected SDOH categories, among which some stressful life events were mentioned: homelessness, domestic violence, and others. However, as these publications considered broad SDOH domains, we couldn’t extract specific workflows that would be related to stressful life events. Some of the general SDOH-related clinical interventions discussed in these papers included: referral to trauma recovery [47], sending educational materials to patient [47], alerts and triggers [43, 48], panel management [43], automatic and manual referrals to third party agencies [43, 45, 49], risk adjustments [50], modification of clinical practices [43, 49], and adjusted provider recommendations [43, 49].

Table 1 provides a summary of reviewed citations. The characteristics for individual citations in table format are provided in Supplementary Appendix S5. For some of the citations the table also includes additional extracted characteristics, such as dependent and independent variables in statistical studies, types of surveys used to capture events, NLP tools used, and relevant information from results, discussion and conclusion sections of reviewed citations.

## Discussion

Stressful life events are a distinct concept from SDOH, being more intrapersonal or interpersonal experiences than external factors. In this review, we found a limited number of citations related to stressful life events and EHR documentation, suggesting this is an area of research with substantial gaps. The obvious explanation for the gaps is that information about life events is not captured adequately in EHR. Existing medical coding systems like ICD, LOINC and SNOMED CT can benefit from enrichment of their stressful life events inventories [95]. At the same time improvement of NLP tools for parsing clinical notes to include a broader spectrum of life events could facilitate the research, and existing SDOH screening tools could be modified to foster collection of such events.

While the study found a limited number of stressful life events in EHR, the area is evolving, and we are likely to see more publications. The ongoing work to implement United States Core Data for Interoperability (USCDI) – a set of standards that EHR vendors must follow when developing software to insure its interoperability [96] – is important for the future reporting of stressful life events in EHR. The current version of USCDI v1, which certified EHRs were required to support by December 31, 2022, doesn’t include stress measures. While further versions of USCDI include stress measures, they seem to be (as indicated by a letter of support in the comments section of USCDI v2 web page [97]) centered around the PRAPARE 1-item stress assessment (“Stress is when someone feels tense, nervous, anxious, or can’t sleep at night because their mind is troubled. How stressed are you?”), which in our opinion may be inadequate to differentiate stressful life events from daily stressors. In fact, many citations, reviewed in this study, relied on this single question, which made it difficult to understand what type of stress or stressful events authors considered. The Gravity Project initiative [98, 99] is working to expand USCDI to include additional stress measures such as minority stress, which sounds promising, while there are indications that National Association of Community Health Centers is working to include stressful life events into USCDI v4 [100].

This study showed that there is significant subjectivity in defining stressful life events which may prevent collection of such events using predefined lists. Previous research shows that even life events which head the list of the Social Readjustment Scale, such as divorce, have a significant interindividual variability and can improve well-being in some instances [101, 102]. To address the problem of screening for stressful life events, it may be beneficial to assess information about recent (12 months or less) life events using a single question on adverse life events with a more detailed approach for distant life events [4].

Alternative screening approaches also need to be considered. For instance, artificial intelligence technologies like chatbots could facilitate consistent data collection about life events and other important health-related aspects of people’s lives in a non-intrusive manner, for example, during waiting time in the clinic. Querying external data sources like population registry, social media, census, police, and financial records for stressful events is feasible, especially if such modules are developed by EHR software vendors. All these approaches require careful workflow planning to ensure that sensitive life details of a patient and their family are protected. One possible solution could be that a particular life event that a person experienced is hidden from most health professionals, and only a general alert flag is set (“Recent negative/traumatic life situation experienced”). This may prompt a dialog between the care provider and the patient so that the latter can provide more details if they feel inclined to.

The need to maintain differential privacy to protect victims is likely to be one of the reasons why there may be specific challenges of the EHR to manage stressful life events. Randell et al. [103] describe data from qualitative interviews of providers to highlight risk scenarios in the setting of the experience of intimate partner violence (IPV). Some disclosures of stressful events in EHRs (such as loss of job or economic difficulties) may be stigmatizing, while others may actually place patients at risk of further harm, for example, if the perpetrators of harm have access to the records. Examples of where disclosures might harm patients include IPV [104] and loss of pregnancy [105].

Clinical utility of stressful life events was mostly represented by papers on decision support systems for identification of potential child abuse – an area which has a well-established tradition of clinical practice. This suggests that new workflows addressing other types of stressful life events in EHR are yet to be defined.

### Limitations

One limitation of this scoping review lies in the definition of stressful life events. Some events mentioned in the studies reviewed, such as household moves and planned immigration (as opposed to forced immigration), were considered normative by our team and were excluded from review, although these events can be very stressful for some family members.

By adding minority stressors, such as discrimination and acculturative stress, we may have shifted the definition of stressful life event as a discrete and acute stressful episode in life to encompass daily stressors, however we think that a more inclusive approach to minority stress doesn’t contradict the overall finding of the review – stressful life events lack systematic representation in EHR. Engagement with diverse communities would be helpful to create a list of stressful events affecting minority populations.

We also excluded citations which mention stressful physical conditions identified by corresponding diagnostic codes in EHR, such as cancer, severe TBI, pregnancy, and others. While these are often life-changing experiences, we were adding them to review only when authors treated them specifically as stressful life events.

More stressful events could be identified by using a broader or iterative search strategy, including more types of events or by adding domains of SDOH to the search terms.

Another limitation is that we didn’t focus specifically on the types of NLP methods that were used to extract events from EHR, although some information about methods is listed in Supplementary Appendix S5. Comparing a list of methods used could be beneficial in seeing how each type of event can be extracted most efficiently. However, this topic is separate from the importance of stressful life events and their impact. The review does not assess how exactly life events storage was implemented in EHR software as most studies did not include sufficient details on this topic.

## Conclusions

Stressful life events are an important issue that complements work on SDOH. More data is needed on how to best capture these events in patients’ health record and/or to extract such events from text descriptions in clinical notes, and how to best intervene to support patients experiencing stressful events in clinical settings. The standards may push the idea (management of stressful events) forward. Lack of vendor support for representation of these concepts probably is a driving factor. USCDI v2 and beyond offer the prospect of standardized representations that could drive future applications.

## Figures and Tables

**Table 1.** Summary of review findings

	Count	Percent from all citations (N=70)

EHR-based statistical studies, in which one of the variables is stressful life event [35, 38, 39, 42, 46, 51–81]	36	51.4 %
Among these studies: stressful events are taken from external surveys instead of EHR [42, 46, 52, 54, 56–58, 61, 62, 64, 65, 69, 74, 76, 77, 79, 81]	17	24.3 %

Citations that use structured field in EHR for storage of stressful life event [34, 43, 45, 47, 48, 50, 53, 54, 59, 60, 66–68, 70–72, 80, 82–88]	24	34.3 %

Citations that use NLP to extract stressful life events from EHR [24, 51, 63, 72, 73, 89–94]	11	15.7 %

Clinical utility citations, which discuss possible interventions for stressful life events in EHR [43–50, 82, 84–88, 92]	15	21.4 %

Citations discussing privacy, and other issues of storing stressful life events in EHR [4, 12, 21, 36, 37]	5	7.1 %

## Data Availability

The data underlying this article are available in the article and in its online supplementary material.
